# Supplementary material for: Co-Occurrence of G6PD Deficiency and SCT among Pregnant Women Exposed to Infectious Diseases
Source: J Clin Med. 2023 Aug 2;12(15):5085. doi: 10.3390/jcm12155085 (PMC10419962; doi:10.3390/jcm12155085)
Supplement: Supplementary file 1 [file jcm-12-05085-s001.zip › jcm-2299493-supplementary.pdf]

Table S1: Multinomial logistic regression analysis of infections, anemia and sickling status with G6PDd

Conditions	Outcomes	B	Standard Error	Exp (B) /AOR	95% CI AOR	*P-value*
Malaria	Negative	0.057	0.198	0.95	0.64-1.39	0.775
	Positive	0[b]				
Syphilis	Negative	0.017	0.741	0.98	0.23-4.20	0.982
	Positive	0[b]				
HIV	Negative	0.051	0.468	0.95	0.38-2.38	0.913
	Positive	0[b]				
Hepatitis B	Negative	0.101	0.285	1.11	0.63-1.94	0.723
	Positive	0[b]				
Anaemia	Negative	0.037	0.104	1.04	0.85-1.27	0.719
	Positive	0[b]				
Sickling status	Negative	0.459	0.181	1.58	1.11-2.26	0.011
	Positive	0[b]				

The reference category is Positive; b: parameter is set to zero because it considered redundant
B: regression coefficient.
Exp (B): exponentiation of B, which is the same as AOR: Adjusted odds ratio.
95% CI: 95% confidence interval
P-value: analyzed by multinomial logistic regression analyses, and considered significant at < 0.05 (2 tailed).

Table S2: Distribution of Demographic and clinical information among G6PDd women

Variable	Sub-group	Frequency	Percentage (%)
	Demographic and obstetric information		
Age, years (n=726)	<18	6	0.8
	18-25	203	28.0
	26-30	282	38.8
	31-40	232	32.0
	40+	3	0.4
Gravidity (n=726)	1-2	357	49.2
	3-4	251	34.6
	4+	118	16.3
Parity (n=726)	0	200	27.5
	1-2	357	49.2
	3-4	133	18.3
	4+	36	5.0
	Anemia		
Anemia (n=726)	Negative	377	51.9
	Positive	349	48.1
